# Validation of the Russian Version of the MoCA Test as a Cognitive Screening Instrument in Cognitively Asymptomatic Older Individuals and Those With Mild Cognitive Impairment

**DOI:** 10.3389/fmed.2020.00447

**Published:** 2020-08-13

**Authors:** Tamar Freud, Anna Vostrikov, Tzvi Dwolatzky, Boris Punchik, Yan Press

**Affiliations:** ^1^Department of Family Medicine, Siaal Research Center for Family Medicine and Primary Care, Faculty of Health Sciences, Ben-Gurion University of the Negev, Be'er Sheva, Israel; ^2^Geriatric Unit, Rambam Health Care Campus, Haifa, Israel; ^3^Ruth and Bruce Rappaport Faculty of Medicine, Technion—Israel Institute of Technology, Haifa, Israel; ^4^Unit for Community Geriatrics, Division of Health in the Community, Ben-Gurion University of the Negev, Be'er Sheva, Israel; ^5^Department of Geriatrics, Soroka Medical Center, Be'er Sheva, Israel

**Keywords:** MoCA, mild cognitive impairment, Russian version, Israel, old age

## Abstract

**Background:** Cognitive impairment is a common condition in older people, and age-related cognitive symptoms may progress to Mild Cognitive Impairment and Dementia. Physical exercise and cognitive training may be useful in maintaining cognitive function, and those developing impaired cognitive function should be advised to plan for the future. The MoCA test is a useful cognitive screening instrument, but the Russian version of this test has not yet been validated. The aim of the present study was to validate the Russian version of the MoCA test.

**Methods:** The study population included 160 residents of Israel aged 65 years and older with Russian as their mother tongue, 80 of whom were cognitively asymptomatic (AC) and 80 with a clinical diagnosis of MCI. All participants underwent cognitive screening using the Russian version of the MoCA test (MoCA-Ru) as well as evaluation by means of a validated computerized cognitive assessment battery (Neurotrax).

**Results:** The mean age of the study population was 78 ± 6.6 years and 123 (76.9%) were women. The MoCA-Ru score was higher in the AC group than in those with MCI (24.3 ± 3.74 vs. 20.2 ± 3.07, *P* < 0.0001). At a cutoff value of ≥25, sensitivity was 0.99 and specificity 0.54, with area under the curve (AUC) of 0.81.

**Conclusions:** We found the Russian language version of the MoCA test to be a useful cognitive screening instrument for older people with mild cognitive impairment.

## Introduction

As the population ages, the number of older people with cognitive decline is increasing. The transition from normal cognitive aging to mild cognitive impairment (MCI) and subsequent dementia has been well-described (1).

MCI (also termed mild neurocognitive disorder according to DSM 5) (2) is a syndrome in which a person experiencing cognitive symptoms is found to have objective cognitive impairment in one or more domains. While there may be minimal difficulties in instrumental activities of daily living, basic activities of daily living are preserved (3, 4). Early diagnosis of MCI allows for providing advice regarding the possible benefits of interventions, such as cognitive training (5), aerobic exercise (6), and planning for the future.

The prevalence of MCI increases with advancing age. The diagnosis of MCI is clinical and is based on Consensus Criteria (7). The determination of cognitive impairment is based on neuropsychological testing designed to evaluate the main cognitive areas affected by age-related cognitive decline (4). There is a clear need to develop valid screening tests to ascertain whether cognitive symptoms require further evaluation by neuropsychological testing.

The Montreal Cognitive Assessment (MoCA) Test developed by Nasreddine et al. (8) has been validated as a reliable cognitive screening test in the older population. This test, which takes only about 10 min to complete, was designed to be effective as a screening instrument for MCI. It has been translated into many languages, and the official website of MoCA includes over 50 translated versions. Some of these translations have been validated, for example those translated to Chinese (9–18), Korean (19), Japanese (20), Dutch (21), Spanish (22), Italian (23) Portuguese (24, 25), Turkish (26), Polish (27), Georgian (28), Arabic (29, 30) and Hebrew (31). A comparison of the findings of these validation studies will be presented later in the Discussion section.

The Russian language is the 7th most common spoken language in the world, with about 260 million people speaking the language globally, and about 150 million native Russian speakers (https://en.wikipedia.org/wiki/Russian_language). Over one million of the 9 million residents of Israel speak Russian. About 20.9% of citizens aged 65 years and older in Israel define Russian as their mother tongue, and Russian is the most common native language in Israel in this age group (32, 33).

To the best of our knowledge the MoCA questionnaire has not yet been validated in Russian. The aim of the present study was to validate the Russian version of MoCA-Ru in a population of Israelis aged 65 years of age and older who define Russian as their native tongue.

## Methods

### Study Population

The study population included 160 participants aged 65 years and older with Russian as their mother tongue, 80 with a diagnosis of MCI according to Consensus Criteria (7) and 80 cognitively asymptomatic controls (AC) based on self-report. According to the Consensus Criteria (7), MCI was diagnosed when “(a) the person is neither normal nor demented; (b) there is evidence of cognitive deterioration shown by either objectively measured decline over time and/or subjective report of decline by self and/or informant in conjunction with objective cognitive deficits; and (3) activities of daily living are preserved and complex instrumental functions are either intact or minimally impaired.”

Data on the 80 MCI patients were collected from the medical records of the Comprehensive Geriatric Assessment Unit of the Clalit Health Services in Be'er Sheva. In this Unit, frail community-dwelling older individuals who reside in the Be'er Sheva region undergo clinical geriatric assessment. The multi-disciplinary staff of the Unit include geriatricians, a nurse, an occupational therapist, a social worker and a dietician. The comprehensive geriatric assessment routinely includes cognitive and functional assessments.

The AC group was a convenience sample recruited by one of the investigators (AV) from a community clinic in Be'er Sheva. Based on self-reporting of symptoms, inclusion criteria for this group were: (a) no cognitive symptoms suggesting cognitive decline, and (b) no complaint relating to cognition-related difficulties in instrumental activities of daily living (IADL). In both groups, individuals with acute medical conditions, as well as those with bipolar disorder, depression, or schizophrenia, were excluded from the study. All participants provided their informed consent to take part in the study. The study was approved by the Helsinki Committee of the Meir Hospital (Approval #104/2014C).

### Cognitive Evaluation

All participants underwent cognitive screening using the Russian version of the MoCA test (8), as well as cognitive assessment by means of the Russian version of the Neurotrax computerized battery, which has been validated for MCI in other languages (34).

The MoCA test includes eight parts with a maximum score of 30 (a score of 30 represents the best cognitive state). A score of 26 or above is considered normal in the English language version (8) and in some of the studies in other languages (12, 17, 20, 21, 29, 31). In the present study we used the original version of MoCA in Russian as translated by Posochin O.B. and Smirnov A.J. (https://www.mocatest.org/wp-content/uploads/2015/tests-instructions/MoCA-Test-Russian_2010.pdf).

The Neurotrax computerized cognitive assessment battery does not require that the subject has previous computer experience and uses standard neuropsychological tests that were adapted for computer use. The results are grouped into cognitive domains, including memory, attention, visuospatial and executive functions, and a composite score for global cognitive function is calculated. Results are corrected for age and education level according to database norms, with a mean score for each domain being 100, and normal being within one standard deviation (15 points). The test takes about 45 min to administer and requires the use of a mouse and number keyboard keys. It has been translated into various languages, including Russian (http://www.neurotrax.com).

### Sample Size

The sample size of 80 in each group was calculated with α = 0.05 and a power of 80% between MoCA and Neurotrax. It was calculated using the Epinfo program 6 (Statcal). We aimed to recruit ninety individuals to each group, anticipating a dropout rate of ~15%.

### Statistical Analyses

The data were analyzed using the Receiving Operating Characteristic (ROC) method to test the capacity of each research instrument (MoCA compared to Neurotrax as the gold standard) to distinguish between patients with MCI and cognitively asymptomatic older participants. Sensitivity, specificity, positive and negative predictive values and the Youden Index (Sensitivity + Specificity −1) values were computed for various MOCA-Ru cutoff points (≥22, 23, 24, 25, 26). In addition, an ANOVA test was performed to compare continuous variables, and chi square or Fisher exact tests were performed for categorical variables. Statistical significance was set at *P* < 0.05.

## Results

### Socio-Demographic Characteristics

The mean age of the study population was 78 ± 6.6 years and 123 (76.9%) were women. On average the participants had immigrated to Israel 20.1 ± 8.8 years earlier. Only 15 participants (9.4%) had <10 years of education, 52 (32.5%) had 10 years of education and 93 (58.1%) had more than 10 years of education. The socio-demographic data of the two study groups are presented in Table 1. The AC group were older and had lived in Israel for a longer period.

**Table 1:** Sociodemographic characteristics of the study population.

	**AC (*N* = 80)**	**MCI (*N* = 80)**	***P***
	***N* (%)**	***N* (%)**	
**GENDER**			
Female	62 (77.5)	61 (76.3)	1.000
**AGE (YEARS)**			
Mean ± SD	80.1 ± 7.1	75.9 ± 5.3	<0.0001
Range	65-95	65-88	
**EDUCATION**			
<10 years	11 (13.8)	4 (5.0)	0.056
10 years	29 (36.2)	23 (28.8)	
More than 10 years	40 (50.0)	53 (66.2)	
**YEARS IN ISRAEL**			
Mean ± SD	23.9 ± 7.8	16.4 ± 8.1	<0.0001
Range	9-70	1-38	

*AC, asymptomatic controls; MCI, mild cognitive impairment*.

### Score Distributions for MoCA-Ru and Neurotrax

The overall score for MoCA-Ru was higher in the AC group (24.3 ± 3.74 vs. 20.2 ± 3.07, *P* ≥ 0.0001). This difference was true for all the test components except for Memory and Language 2 (Table 2). The internal consistency of the MoCA-Ru was limited, with a Cronbach's α = 0.65.

**Table 2:** Comparison of the components of the MoCA-Ru, by group.

	**AC (*N* = 80)**	**MCI (*N* = 80)**	***P***
	**Mean ± SD**	**Mean ± SD**	
Trail B	0.49 ± 0.50	0.18 ± 0.38	<0.0001
Cube	0.74 ± 0.44	0.58 ± 0.50	0.031
Clock	2.49 ± 0.76	2.13 ± 0.85	0.005
Naming	2.96 ± 0.25	2.81 ± 0.45	0.010
Memory	1.91 ± 1.76	1.26 ± 1.26	0.008
Attention 1	1.75 ± 0.49	1.74 ± 0.52	0.876
Attention 2	0.94 ± 0.24	0.79 ± 0.41	0.006
Attention 2	2.60 ± 0.81	2.33 ± 0.93	0.047
Language 1	1.90 ± 0.34	1.24 ± 0.75	<0.0001
Language 2	0.29 ± 0.48	0.24 ± 0.43	0.489
Abstraction	1.87 ± 0.40	1.19 ± 0.83	<0.0001
Orientation	5.95 ± 0.27	5.69 ± 0.67	0.001
**MOCA-Ru total score**	24.3 ± 3.74	20.2 ± 3.07	<0.0001

*AC, asymptomatic controls; MCI, mild cognitive impairment; MoCA, Montreal Cognitive Assessment*.

The global score of the Neurotrax battery was significantly higher in the AC group than in the MCI group (84.3 ± 10.7 vs. 79.9 ± 8.9, respectively, *P* = 0.005), but the only domain with a significant difference was Memory [86.7 ± 14.5 (AC) vs. 84.3 ± 10.7 (MCI), *P* < 0.0001]. Only six of the 80 participants in the AC group had a Neurotrax global score ≥100. For that reason, in a *post hoc* analysis, a sub-group of 93 participants was created including 52 participants from the MCI group with a global Neurotrax score ≤ 85 (more than one standard deviation below normal) and 41 from the AC group with a global score above 85 (more than one standard deviation below normal). The calculation of power for this sample was 98%. In this sub-group analysis the MoCA-Ru score was 25.66 ± 3.5 in the AC group and 19.4 ± 3.0 in the MCI group (*P* < 0.0001).

### Validity of the MoCA-Ru for Detecting MCI

The sensitivity, specificity, positive, and negative values and the Youden Index for MoCA-Ru were calculated for the entire study population, as well as for the 93 participants in the selected sub-group (Table 3).

**Table 3:** Sensitivity, specificity, positive, and negative predictive values and Youden Index for the MoCA-Ru test, with different cutoffs.

**Cutoff: normal value**	**Sensitivity**	**Specificity**	**PPV**	**NPV**	**Youden Index**
**UNSELECTED STUDY POPULATION (*****N*** **=** **160)**					
≥26	1	0.4	0.63	1	0.4
≥25	0.99	0.54	0.68	0.98	0.53
≥24	0.83	0.63	0.69	0.78	0.45
≥23	0.73	0.72	0.72	0.71	0.46
**SELECTED SUB-GROUP (*****N*** **=** **93)**					
≥26	1	0.63	0.78	1	0.63
≥25	0.98	0.73	0.83	0.97	0.71
≥24	0.91	0.78	0.85	0.87	0.69
≥23	0.85	0.81	0.85	0.81	0.66

*PPV, positive predictive value; NPV, negative predictive value; MoCA, Montreal Cognitive Assessment*.

In the unselected population of 160 participants the normal optimal cut-off for the MoCA-Ru with the highest Youden Index was ≥25, with a sensitivity of 0.99, but with a low specificity of 0.54. In the selected group of 93 participants the optimal cut-off for MoCA-Ru was similarly ≥25, with higher specificity (0.73) and similar sensitivity (0.98).

**Figure 1:**
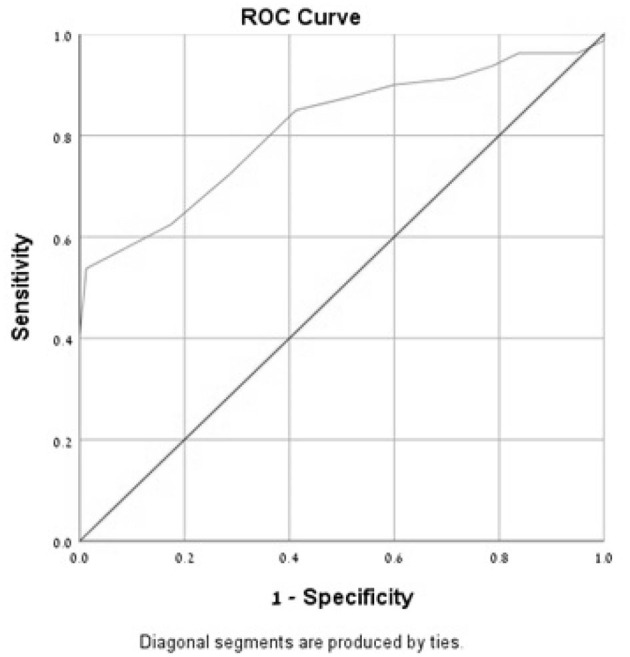
Receiver-operator characteristics curves (ROC) of the MoCA-Ru test in an unselected study population (cut-off 25).

**Figure 2:**
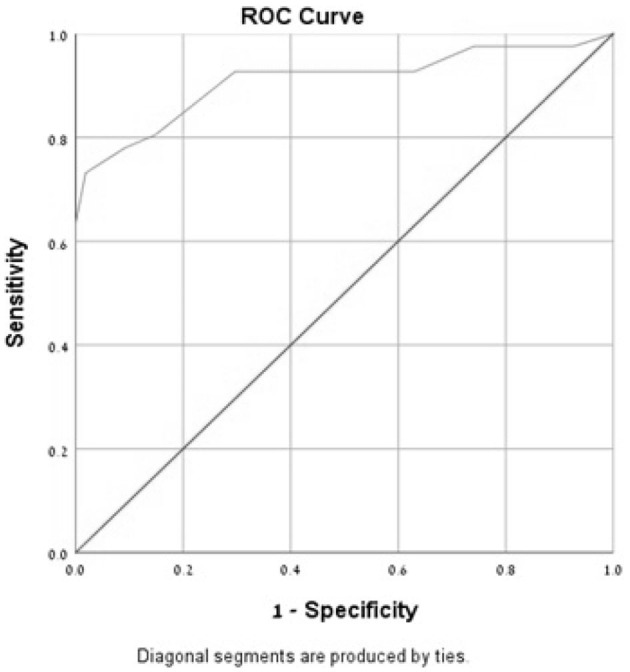
Receiver-operator characteristics curves (ROC) of the MoCA-Ru test in a selected study population (cut-off 25).

In the ROC curve analysis of the MoCA-Ru score, the area under the curve (AUC) was 0.81 for all subjects and 0.91 for the selected population, confirming that the test has a good discriminating capacity for differentiating participants with MCI from asymptomatic controls (Figures 1, 2).

## Discussion

The results of the present study show that the MoCA-Ru test has good discriminating capacity for differentiating participants with MCI from cognitively asymptomatic controls. In contrast to the original English version of the MoCA (8) in which the normal cut-off value was set at ≥26, the suggested cut-off value in our study is ≥25.

Table 4 summarizes findings from previous studies that have evaluated the validity of MoCA among populations from various countries in different languages. It can be seen that the cut-off value varies between studies. A meta-analysis that included nine studies (37) showed that a cut-off of ≥23 had the best diagnostic accuracy.

**Table 4:** Comparison of versions of the MoCA, by language.

**Language**	**Country**	**First author**	**Year of publication**	**Normal cut-off**	**Sensitivity**	**Specificity**	**AUC**	**Cronbach's alpha**
Arabic	Egypt	Rahman and El Gaafary (29)	2009	≥26	0.923	0.857	NR	0.83
Chinese	China	Zhao et al. (18)	2011	≥24	0.772	0.901	0.882	0.889
Chinese	China	Lu et al. (11)	2011	Adjusted for education: Illiterate ≥ 14; 1–6 years ≥ 20; ≥ 7 years ≥ 25	0.805	0.825	0.899	0.85
Chinese	Hong Kong	Yeung et al. (16)	2014	≥22	0.828	0.735	0.829	0.767
Chinese	Singapore	Ng et al. (13)	2015	GRP sample= ≥23	0.65	0.55	0.63	NR
				NNI sample= ≥29	0.64	0.36	0.65	NR
Chinese	China	Yu et al. (17)	2013	≥22	0.687	0.639	0.71	0.88
Chinese	Singapore	Ng et al. (12)	2013	≥26	0.96	0.3		NR
Chinese	Hong Kong	Chu et al. (35)	2014	≥23	0.78	0.73	0.85	0.85
Chinese	Singapore	Dong et al. (9)	2013	≥20	0.8	0.92	0.94	NR
Chinese	China	Hu et al. (10)	2013	≥27	0.92	0.85	0.928	0.867
Chinese	China	Tan et al. (14)	2014	Adjusted for age:			0.937	0.953
				60–79 years ≥26	0.858	0.854		
				80–89 years ≥25 ≥ 90 years ≥24	0.853	0.963		
					0.903	0.969		
Chinese	Taiwan	Tsai et al. (15)	2016	≥24	0.88	0.74	0.91	NR
Dutch	Nederland	Thissen et al. (21)	2010	≥26	0.72	0.73	NR	0.69
English	USA	Goldstein et al. (36)	2014	≥25	0.95	0.63	0.79	NR
English	Canada	Nasreddine et al. (8)	2005	≥26	0.9	0.87	NR	0.83
Georgian	Georgia	Janelidze et al. (28)	2017	≥22	1.0	0.69	0.88	0.92
Hebrew	Israel	Lifshitz et al. (31)	2012	≥26	0.946	0.763	0.963	NR
Italian	Italy	Bosco et al. (23)	2017	≥20	0.692	0.585	0.685	NR
Japanese	Japan	Fujiwara et al. (20)	2010	≥26	0.93	0.89	0.95	0.74
Korean	South Korea	Lee et al. (19)	2008	≥23	0.89	0.84	0.94	0.86
Polish	Poland	Magierska et al. (27)	2012	≥25	0.809	0.54	0.74	NR
Portuguese	Portugal	Freitas et al. (24)	2013	≥22	0.81	0.77	0.856	0.9
Portuguese	Brazil	Memória et al. (25)	2013	≥25	0.81	0.77	0.82	0.75
Russian	Israel	Present study		≥25	0.99	0.54	0.81	0.65
Spanish	Colombia	Gil et al. (22)	2015	≥23	0.89	0.798	0.93	0.846
Turkish	Turkey	Kaya et al. (26)	2014	Adjusted for education:			0.846	0.81
				≤ 5 years≥18	0.67	0.83		
				6-12 years≥21	0.73	0.85		
				≥12 years≥23	0.81	0.86		

*GRP, Gerontology Research Program; NNI, National Neuroscience Institute; NR, Not reported*.

A clear advantage of our study is that it is the first study to evaluate the validity of MoCA in a Russian speaking population. However, our study has clear limitations, which raise the question as to the generalizability of the study results. An important limitation is that the methodology of validation of the MoCA that we used in our study differed from that used for validation in a number of other languages. The majority of studies (9–12, 14–20, 22, 24–28, 30) included subjects with either MCI or dementia as well as controls. In contrast, as is the case with some of the other reported studies (13, 23, 29, 31), our study compared only subjects with MCI to a control group. However, it is important to note that in the original validation study of the MoCA, the investigators found similar results for both the MCI and dementia groups (8).

Another major limitation of our study is the choice of a group of controls who were cognitively asymptomatic based on self-report alone. While the subjects were diagnosed with MCI based on clinical evaluation, the asymptomatic control group did not undergo formal cognitive evaluation prior to inclusion in the study. This is in contrast to many of the studies where the control group included those with “normal cognition” based on an absence of cognitive symptoms as well as preserved function on cognitive testing (9–20, 31). Nevertheless, some studies did include a group of asymptomatic controls with normal cognitive screening (23, 28–30) but no formal cognitive evaluation.

It is important to note that the Neurotrax global score (84.3 ± 10.7) of the control group raises the possibility that some of the participants indeed had cognitive impairment beyond the level anticipated for their age and education level. We thus performed a sub-group analysis to include only cognitively normal controls based on Neurotrax findings compared to those with MCI. In this analysis the normal cut-off for the MoCA-Ru tests remained at ≤ 25, with improved specificity, supporting the validity of our findings. The internal consistency of the MoCA-Ru in our study was lower than in most of the studies listed in Table 4.

This convenience sample of asymptomatic controls was also older than our subjects with MCI. However, it is important to emphasize that Neurotrax scores are corrected for age and education level. Also, since the prevalence of MCI increases with age, the older age of the controls actually strengthens our findings.

Many of the studies evaluating different language versions of the MoCA utilized neuropsychological batteries (9, 11, 14, 16, 19, 22, 24, 26) while others used the MMSE with the Clinical Dementia Rating Scale (CDR) Scale (12, 15, 17, 27, 30) or even the MMSE alone (23, 28). In our study we used the Neurotrax computerized cognitive assessment battery, which was also used to validate the Hebrew version of the MoCA (31), and which has been shown to be reliable for determining the presence of MCI (34).

Our optimal normal cut-off level was ≥25, which is slightly lower than that of ≥26 found by Nasreddine et al. (8). A possible explanation for this finding may be related to the age of the participants. In our study, the age of the participants was relatively high compared to other studies in this field (Table 4). There is no consensus as to the effect of age on the results of the MoCA test. In some studies, an association with age was found (9, 11–14, 26), while others did not find such an association (10, 22, 35). The association between MoCA and education level has been described (9–14, 16, 22, 26, 35), with a positive association found between education levels and the MoCA score (25). In the present study <10% of the participants had an education level lower than 10 years (which is equivalent to high school education in the former Soviet Union). On comparing our study to those in which the study population was well-educated, the optimal normal cut-off was ≥26, as found in the study by Nasreddine et al. (8) Thus, for example, in the study conducted in Spanish in Colombia by Gil et al. (22) where the normal cut-off was ≥26, the sensitivity of MoCA was 0.99 and the specificity was 0.52, but when the cut-off was set at ≥23 the sensitivity dropped to 0.89 while the specificity increased to 0.79. In the study by Goldstein et al. (36) among African Americans the mean education level was 13.4 years in the MCI group and 10.9 in the control group. At a normal cut-off of ≥26 the sensitivity of MoCA was 1.0 with a specificity of 0.4, while at ≥25 the sensitivity was 0.95 and the specificity 0.63.

In a study from Georgia (28), the mean education level was 11.5 years in the MCI group and 11.6 years in the control group. At the recommended cut-off value of ≥26 the sensitivity of MoCA was 1.0 and the sensitivity was 0.44, but when the cut-off was set at ≥22 the specificity increased to 0.69 without any change in sensitivity (1.0). It is important to note that it is appropriate to compare the results of the Georgian study with those of the present study not only since the populations in both studies were well-educated, but also because the two populations were composed of former residents of the USSR who received an education in schools with similar curricula.

A clear advantage of our study is that it is the first study to evaluate the validity of MoCA in a Russian speaking population. However, this study has clear limitations. The major limitation of the study, which was already discussed in detail above, was that the participants in the control group did not undergo a comprehensive cognitive, affective, and functional assessment, and that their eligibility was based on self-reported normal cognitive and instrumental function. We can thus not exclude the possibility that subjects in the control group may indeed have suffered cognitive impairment. As well, the participants in the control group were recruited from community clinics and not at random. All these factors raise the question as to the generalizability of the study results. Several variables were not collected in the control group. For example, we do not have data on chronic co-morbidity or medical treatment for these participants, so we could not compare the two study groups beyond basic socio-demographic details.

Another limitation of the present study is related to the optimal cut-off, which was determined on the basis of the data analyses. This way of determining the optimal cut-off can increase the risk for bias (38) and could lead to an overestimation of sensitivity and specificity, especially in small studies (39).

In conclusion, we found the Russian language version of the MoCA test to be useful as a screening tool for MCI. Further studies should aim to further validate this instrument and to determine the optimal normal cut-off value for MoCA-Ru in the older population with impaired cognitive function.

## Data Availability Statement

The datasets generated for this study are available on request to the corresponding author.

## Ethics Statement

The studies involving human participants were reviewed and approved by Helsinki Committee of the Meir Hospital (Approval #104/2014C). The patients/participants provided their written informed consent to participate in this study.

## Author Contributions

TF designed the study, was responsible for the statistical design of the study, and for carrying out the statistical analysis. AV designed the study, collected the data, and assisted with writing the article. TD and BP designed the study and assisted with writing the article. YP designed the study and wrote the article. All authors contributed to the article and approved the submitted version.

## Conflict of Interest

The authors declare that the research was conducted in the absence of any commercial or financial relationships that could be construed as a potential conflict of interest.

## References

[1] Campbell NL Unverzagt F LaMantia MA Khan BA Boustani MA. Risk factors for the progression of mild cognitive impairment to dementia. Clin Geriatr Med. (2013) 29:873–93. 10.1016/j.cger.2013.07.009 24094301 PMC5915285

[2] Association AP. Diagnostic and Statistical Manual of Mental Disorders: DSM-5. Washington, DC (2013).

[3] Gauthier S Reisberg B Zaudig M Petersen RC Ritchie K Broich K. Mild cognitive impairment. Lancet. (2006) 367:1262–70. 10.1016/S0140-6736(06)68542-5 16631882

[4] Petersen RC Caracciolo B Brayne C Gauthier S Jelic V Fratiglioni L. Mild cognitive impairment: a concept in evolution. J Int Med. (2014) 275:214–28 10.1111/joim.12190 24605806 PMC3967548

[5] Butler M McCreedy E Nelson VA Desai P Ratner E Fink HA Does cognitive training prevent cognitive decline?: a systematic review. Anna Int Med. (2018) 168:63–8. 10.7326/M17-1531 29255842

[6] Song D Yu DSF Li PWC Lei Y. The effectiveness of physical exercise on cognitive and psychological outcomes in individuals with mild cognitive impairment: a systematic review and meta-analysis. Int J Nursing Stud. (2018) 79:155–64. 10.1016/j.ijnurstu.2018.01.002 29334638

[7] Winblad B Palmer K Kivipelto M Jelic V Fratiglioni L Wahlund LO. Mild cognitive impairment–beyond controversies, towards a consensus: report of the International Working Group on Mild Cognitive Impairment. J Int Med. (2004) 256:240–6. 10.1111/j.1365-2796.2004.01380.x 15324367

[8] Nasreddine ZS Phillips NA Bedirian V Charbonneau S Whitehead V Collin I The montreal cognitive assessment, MoCA: a brief screening tool for mild cognitive impairment. J Am Geriatr Soc. (2005) 53:695–9. 10.1111/j.1532-5415.2005.53221.x 15817019

[9] Dong Y Yean Lee W Hilal S Saini M Wong TY Chen CL. Comparison of the montreal cognitive assessment and the mini-mental state examination in detecting multi-domain mild cognitive impairment in a Chinese sub-sample drawn from a population-based study. Int Psychogeriatr. (2013) 25:1831–8. 10.1017/S1041610213001129 23870281

[10] Hu JB Zhou WH Hu SH Huang ML Wei N Qi HL. Cross-cultural difference and validation of the Chinese version of montreal cognitive assessment in older adults residing in Eastern China: preliminary findings. Arch Gerontol Geriatr. (2013) 56:38–43. 10.1016/j.archger.2012.05.008 22698678

[11] Lu J Li D Li F Zhou A Wang F Zuo X. Montreal cognitive assessment in detecting cognitive impairment in Chinese elderly individuals: a population-based study. J Geriatr Psychiatr Neurol. (2011) 24:184–90. 10.1177/0891988711422528 22228824

[12] Ng A Chew I Narasimhalu K Kandiah N. Effectiveness of montreal cognitive assessment for the diagnosis of mild cognitive impairment and mild Alzheimer's disease in Singapore. Singapore Med J. (2013) 54:616–9. 10.11622/smedj.2013220 24276096

[13] Ng TP Feng L Lim WS Chong MS Lee TS Yap KB. Montreal Cognitive Assessment for screening mild cognitive impairment: variations in test performance and scores by education in Singapore. Dementia Geriatr Cogn Disord. (2015) 39:176–85. 10.1159/000368827 25572449

[14] Tan JP Li N Gao J Wang LN Zhao YM Yu BC. Optimal cutoff scores for dementia and mild cognitive impairment of the montreal cognitive assessment among elderly and oldest-old Chinese population. J Alzheimer's Dis. (2015) 43:1403–12. 10.3233/JAD-141278 25147113

[15] Tsai JC Chen CW Chu H Yang HL Chung MH Liao YM. Comparing the sensitivity, specificity, and predictive values of the montreal cognitive assessment and mini-mental state examination when screening people for mild cognitive impairment and dementia in Chinese population. Arch Psychiatr Nurs. (2016) 30:486–91. 10.1016/j.apnu.2016.01.015 27455923

[16] Yeung PY Wong LL Chan CC Leung JL Yung CY. A validation study of the Hong Kong version of Montreal Cognitive Assessment. (HK-MoCA) in Chinese older adults in Hong Kong. Hong Kong Med J. (2014) 20:504–10. 10.12809/hkmj144219 25125421

[17] Yu J Li J Huang X. The Beijing version of the Montreal Cognitive Assessment as a brief screening tool for mild cognitive impairment: a community-based study. BMC Psychiatr. (2012) 12:156. 10.1186/1471-244X-12-156 23009126 PMC3499377

[18] Zhao S Guo C Wang M Chen W Wu Y Tang W. A clinical memory battery for screening for amnestic mild cognitive impairment in an elderly chinese population. J Clin Neurosci. (2011) 18:774–9. 10.1016/j.jocn.2010.07.149 21435882

[19] Lee JY Dong Woo L Cho SJ Na DL Hong Jin J Kim SK. Brief screening for mild cognitive impairment in elderly outpatient clinic: validation of the Korean version of the Montreal Cognitive Assessment. J Geriatr Psychiatr Neurol. (2008) 21:104–10. 10.1177/0891988708316855 18474719

[20] Fujiwara Y Suzuki H Yasunaga M Sugiyama M Ijuin M Sakuma N. Brief screening tool for mild cognitive impairment in older Japanese: validation of the Japanese version of the Montreal Cognitive Assessment. Geriatr Gerontol Int. (2010) 10:225–32. 10.1111/j.1447-0594.2010.00585.x 20141536

[21] Thissen AJ van Bergen F de Jonghe JF Kessels RP Dautzenberg PL. [Applicability and validity of the dutch version of the montreal cognitive assessment. (moCA-d) in diagnosing MCI]. Tijdschrift Voor Gerontologie En Geriatrie. (2010) 41:231–40. 10.1007/s12439-010-0218-0 21229776

[22] Gil L Ruiz de Sanchez C Gil F Romero SJ Pretelt Burgos F. Validation of the montreal cognitive assessment. (MoCA) in Spanish as a screening tool for mild cognitive impairment and mild dementia in patients over 65 years old in Bogota, Colombia. Int J Geriatr Psychiatr. (2015) 30:655–62. 10.1002/gps.4199 25320026

[23] Bosco A Spano G Caffo AO Lopez A Grattagliano I Saracino G. Italians do it worse. Montreal Cognitive Assessment. (MoCA) optimal cut-off scores for people with probable Alzheimer's disease and with probable cognitive impairment. Aging Clin Exp Res. (2017) 29:1113–20. 10.1007/s40520-017-0727-6 28155182

[24] Freitas S Simoes MR Alves L Santana I Montreal cognitive assessment: validation study for mild cognitive impairment and Alzheimer disease. Alzheimer Dis Assoc Disord. (2013) 27:37–43. 10.1097/WAD.0b013e3182420bfe 22193353

[25] Memoria CM Yassuda MS Nakano EY Forlenza OV. Brief screening for mild cognitive impairment: validation of the Brazilian version of the Montreal cognitive assessment. Int J Geriatr Psychiatr. (2013) 28:34–40. 10.1002/gps.3787 22368034

[26] Kaya Y Aki OE Can UA Derle E Kibaroglu S Barak A. Validation of montreal cognitive assessment and discriminant power of montreal cognitive assessment subtests in patients with mild cognitive impairment and alzheimer dementia in turkish population. J Geriatr Psychiatr Neurol. (2014) 27:103–9. 10.1177/0891988714522701 24578463

[27] Magierska J Magierski R Fendler W Kloszewska I Sobow TM. Clinical application of the Polish adaptation of the Montreal Cognitive Assessment. (MoCA) test in screening for cognitive impairment. Neurol Neurochirurg Polska. (2012) 46:130–9. 10.5114/ninp.2012.28255 22581594

[28] Janelidze M Mikeladze N Bochorishvili N Dzagnidze A Kapianidze M Mikava N. Validity of the georgian montreal cognitive assessment for the screening of mild cognitive impairment and Dementia. Am J Alzheimer's Dis Other Dementias. (2017) 32:36–40. 10.1177/1533317516679304 27909150 PMC10852627

[29] Rahman TT El Gaafary MM. Montreal cognitive assessment Arabic version: reliability and validity prevalence of mild cognitive impairment among elderly attending geriatric clubs in Cairo. Geriatr Gerontol Int. (2009) 9:54–61 10.1111/j.1447-0594.2008.00509.x 19260980

[30] Saleh AA Alkholy R Khalaf OO Sabry NA Amer H El-Jaafary S. Validation of montreal cognitive assessment-basic in a sample of elderly egyptians with neurocognitive disorders. Aging Mental Health. (2018) 23: 551–7. 10.1080/13607863.2018.1428936 29424560

[31] Lifshitz M Dwolatzky T Press Y. Validation of the Hebrew version of the MoCA test as a screening instrument for the early detection of mild cognitive impairment in elderly individuals. J Geriatr Psychiatr Neurol. (2012) 25:155–61. 10.1177/0891988712457047 23124009

[32] Dwolatzky T Brodsky J Azaiza F Clarfield AM Jacobs JM Litwin H. Coming of age: health-care challenges of an ageing population in Israel. Lancet. (2017) 389:2542–50. 10.1016/S0140-6736(17)30789-4 28495114

[33] Brodsky J SY Be'er S The Elderly in Israel: Statistical Abstract 2016 (2017).

[34] Dwolatzky T Whitehead V Doniger GM Simon ES Schweiger A Jaffe D. Validity of a novel computerized cognitive battery for mild cognitive impairment. BMC Geriatr. (2003) 3:4. 10.1186/1471-2318-3-4 14594456 PMC270050

[35] Chu LW Ng KH Law AC Lee AM Kwan F. Validity of the cantonese chinese montreal cognitive assessment in Southern Chinese. Geriatr Gerontol Int. (2015) 15:96–103. 10.1111/ggi.12237 24456109

[36] Goldstein FC Ashley AV Miller E Alexeeva O Zanders L King V. Validity of the montreal cognitive assessment as a screen for mild cognitive impairment and dementia in African Americans. J Geriatr Psychiatr Neurol. (2014) 27:199–203. 10.1177/0891988714524630 24614202

[37] Carson N Leach L Murphy KJ. A re-examination of Montreal Cognitive Assessment. (MoCA) cutoff scores. Int J Geriatr Psychiatr. (2018) 33:379–88. 10.1002/gps.4756 28731508

[38] Davis DH Creavin ST Noel-Storr A Quinn TJ Smailagic N Hyde C. Neuropsychological tests for the diagnosis of Alzheimer's disease dementia and other dementias: a generic protocol for cross-sectional and delayed-verification studies. Cochrane Database Syst Rev. (2013) 28:CD010460. 10.1002/14651858.CD010460 25177209 PMC4147664

[39] Leeflang MM Moons KG Reitsma JB Zwinderman AH. Bias in sensitivity and specificity caused by data-driven selection of optimal cutoff values: mechanisms, magnitude, and solutions. Clin Chem. (2008) 54:729–37. 10.1373/clinchem.2007.096032 18258670

